# Real-world effectiveness and safety of risankizumab in patients with plaque psoriasis in whom guselkumab failed recently: A multicenter retrospective study of switching within the interleukin-23 inhibitor class

**DOI:** 10.1016/j.jdin.2023.05.006

**Published:** 2023-05-26

**Authors:** Ahmed Bagit, Khalad Maliyar, Jorge R. Georgakopoulos, Brian Rankin, Alexander Rimke, Asfandyar Mufti, Heather Le, Ronald Vender, Vimal H. Prajapati, Jensen Yeung

**Affiliations:** a Temerty Faculty of Medicine, University of Toronto, Toronto, Ontario, Canada; b Division of Dermatology, Department of Medicine, University of Toronto, Ontario, Canada; c Division of Dermatology, Department of Medicine, University of Calgary, Alberta, Canada; d Dermatology Research Institute, Calgary, Alberta, Canada; e Division of Dermatology, Sunnybrook Health Sciences Centre, Toronto, Ontario, Canada; f Dermatrials Research Inc. and Venderm Consulting, Hamilton, Ontario, Canada; g Section of Community Pediatrics, University of Calgary, Calgary, Alberta, Canada; h Section of Pediatric Rheumatology, Department of Pediatrics, University of Calgary, Calgary, Alberta, Canada; i Skin Health & Wellness Centre, Calgary, Alberta, Canada; j Probity Medical Research, Calgary, Alberta, Canada; k Division of Dermatology, Women’s College Hospital, Toronto, Ontario, Canada

**Keywords:** biologic, efficacy, guselkumab, IL-23 inhibitor, psoriasis, risankizumab, safety

*To the Editor:* The benefit of switching between biologics is being documented by a growing body of evidence in the plaque psoriasis literature.^1^^,^^2^ However, intraclass switching is a new phenomenon, and its real-world effectiveness and safety remain understudied. A pertinent gap in the literature also exists regarding long-term outcomes of switching between the 2 interleukin (IL)-23 inhibitors guselkumab and risankizumab. This study examined the effectiveness and safety of administering risankizumab to patients with plaque psoriasis who have had an inadequate response to guselkumab.

We conducted a multicenter retrospective study of patients aged ≥18 years with moderate-to-severe plaque psoriasis (baseline Physician Global Assessment score of 3 or 4) who received risankizumab following an inadequate response to guselkumab. Responders were those who achieved 90% improvement in baseline Psoriasis Area and Severity Index (PASI90) or Physician Global Assessment scores of 0 (clear) or 1 (almost clear).

The baseline demographics and clinical outcomes are summarized in Table I. At weeks 16 and 52, 69.7% (23/33) and 75% (18/24) of patients, respectively, were considered responders. Additionally, 86.4% (19/22) and 82.4% (14/17) of patients achieved an absolute PASI of ≤2 at weeks 16 and 52, respectively. These results agree with PASI90 outcomes reported in phase 3 randomized controlled trials in which risankizumab was investigated in IL-23–naïve patients (IMMerge: 73.8% and 86.6% at weeks 16 and 52, respectively; UltIMMa-1: 75.3% and 81.9% at weeks 16 and 52, respectively; UltIMMa-2: 74.8% and 80.6% at weeks 16 and 52, respectively).^3^^,^^4^

**Table I:** Baseline demographics and characteristics of patients at the risankizumab treatment initiation baseline visit and the relevant clinical outcomes after 16 and 52 weeks of treatment

Variable	Value
Sex, *n* (%)	
Male	16/33 (48.5)
Female	17/33 (51.5)
Mean age, years ± SD	54.3 ± 14.8
Number of previously failed biologic therapies, mean ± SD	2.39 ± 1.41
Number of days treated with guselkumab ± SD	284.97 ± 376.26
Effectiveness	
Risankizumab responders, *n* (%)	
≥PASI90 or PGA 0/1	
16 wk	23/33 (69.7)
52 wk	18/24 (75)
≥PASI75 or PGA 0/1	
16 wk	24/33 (72.7)
52 wk	18/24 (75)
Mean PASI change	
16 wk	5.31 ± 3.86
52 wk	3.18 ± 4.2
PASI < 1	
16 wk	15/23 (65.2)
52 wk	10/19 (52.6)
PASI ≤ 2	
16 wk	19/22 (86.4)
52 wk	14/17 (82.4)
BSA < 1%	
16 wk	14/27 (51.9)
52 wk	15/21 (71.4)
Safety	
Reported adverse events, *n* (%)	
Thromboembolism	1/24 (4.17)
Injection-site reaction	1/33 (3.03)

*BSA*, Body surface area; *PASI*, Psoriasis Area and Severity Index; *PGA*, Physician Global Assessment.

Among primary guselkumab nonresponders, 60% (9/15) of patients responded to risankizumab at week 16 (Fig 1) and 46.7% (7/15) maintained their responses at week 52. Among secondary guselkumab nonresponders, 77.8% (14/18) responded at week 16 and 22.2% (4/18) maintained their response at week 52. Our findings suggest a lack of correlation between the reason for discontinuing guselkumab and the patients’ response after switching to risankizumab. This is consistent with a previous study conducted by our group that explored the effects of biologic switching between ixekizumab and secukinumab, 2 IL-17A inhibitors^2^; however, this, in part, contradicts findings seen in previous reports of biologic switching.^1^

**Fig 1:**
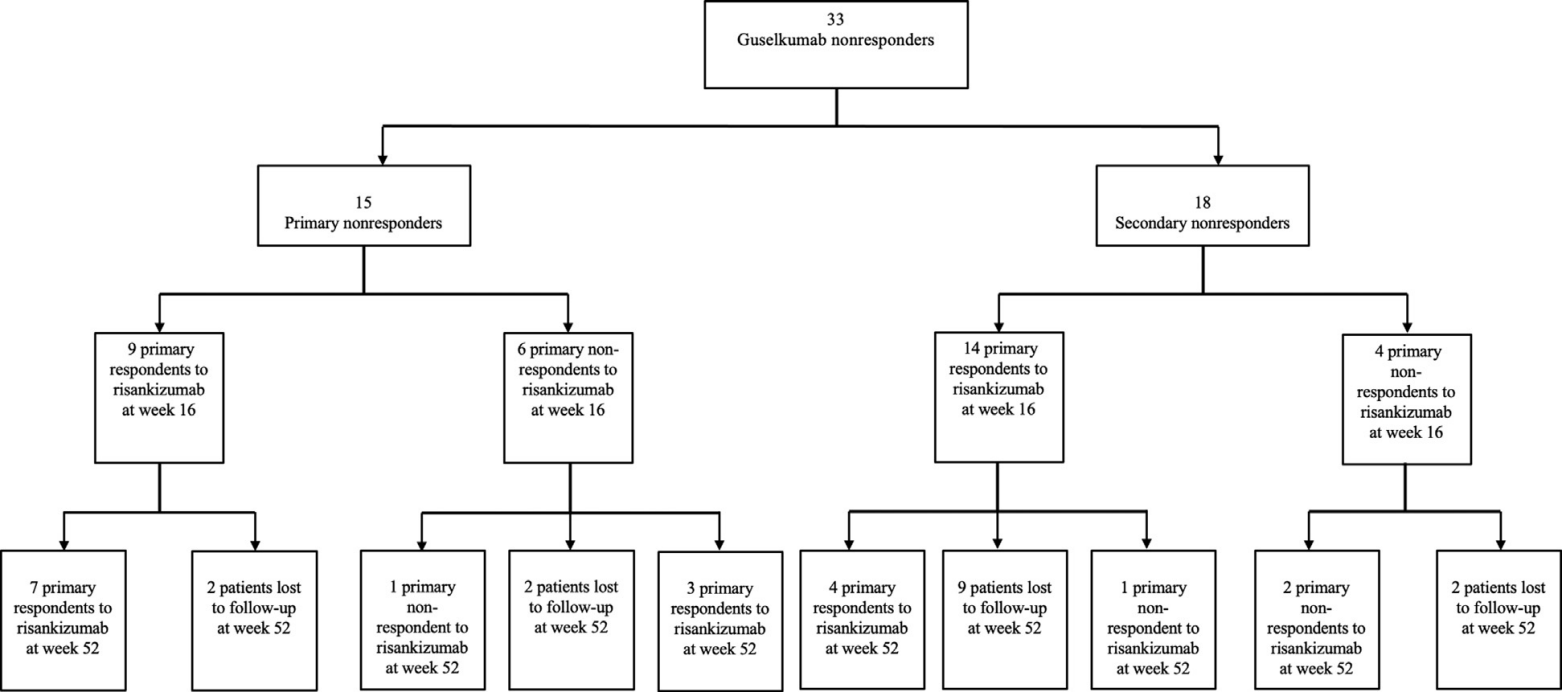
Effectiveness outcomes in 33 patients with moderate-to-severe plaque psoriasis treated with guselkumab followed by risankizumab. Primary nonresponder: did not achieve Psoriasis Area and Severity Index 90 or a Physician Global Assessment score of 0 or 1 at any point during the guselkumab treatment course. Secondary nonresponder: achieved Psoriasis Area and Severity Index 90 or a Physician Global Assessment score of 0 or 1 following 16 weeks of treatment but experienced loss of effectiveness afterwards.

Adverse events (AEs) to risankizumab were uncommon, with 1 AE reported in each of the 16- and 52-week cohorts. Injection-site reaction led to discontinuation of risankizumab in 1 patient prior to 16 weeks; in another patient, a non–life-threatening venous thromboembolism developed between 16 and 52 weeks, with risankizumab being subsequently discontinued because of an unsatisfactory response. Neither of the 2 aforementioned patients reported AEs while on guselkumab.

In summary, we present the largest cohort of patients with long-term follow-up, highlighting the favorable real-world effectiveness and safety of risankizumab for plaque psoriasis in guselkumab-failure patients. The results of our study support the growing body of literature regarding the promising effectiveness and safety profiles of risankizumab, with the added perspective of switching from guselkumab.^5^ Although our clinical experience encourages dermatologists to consider the benefit of IL-23 inhibitor intraclass switching, additional prospective studies with larger sample sizes are required to postulate stronger evidence.

## Conflicts of interest

Dr Yeung has been an advisor, consultant, speaker, and/or investigator for AbbVie, Allergan, Amgen, Arcutis, Astellas, Bausch Health, Boehringer Ingelheim, Bristol Meyers Squibb, Celgene, Centocor, Coherus, Dermavant, Dermira, Forward, Galderma, GlaxoSmithKline, Incyte, Janssen, Kyowa, Leo Pharma, Lilly, Medimmune, Merck, Novartis, Pfizer, Regeneron, Roche, Sandoz, Sanofi Genzyme, SunPharma, Takeda, UCB, Valeant (Bausch Health), and Xenon. Dr Prajapati has served as an investigator for AbbVie, Amgen, Arcutis, Arena, Asana, Bausch Health, Boehringer Ingelheim, Bristol Myers Squibb, Celgene, Concert, Dermavant, Dermira, Eli Lilly, Galderma, Incyte, Janssen, LEO Pharma, Nimbus Lakshmi, Novartis, Pfizer, Regeneron, Reistone, Sanofi Genzyme, UCB, and Valeant and has served as a consultant, advisor, and/or speaker for AbbVie, Actelion, Amgen, Aralez, Arcutis, Aspen, Bausch Health, Boehringer Ingelheim, Bristol Myers Squibb, Celgene, Cipher, Eli Lilly, Galderma, GlaxoSmithKline, Homeocan, Janssen, LEO Pharma, L'Oreal, Medexus, Novartis, Pediapharm, Pfizer, Sanofi Genzyme, SunPharma, Tribute, UCB, and Valeant. Dr Vender received grants/research support from Abbvie, Amgen, Bausch Health, Centocor, Dermira, Dermavant, Galderma, GSK, Leo, Lilly, Takeda, Novartis, Merck, Pfizer, Regeneron, UCB; received honoraria as a speaker for AbbVie, Amgen, Janssen, Galderma, GSK, Leo, Lilly, Merck, Novartis, Pfizer, Bausch-Health, Actelion, Celgene, Cipher, and UCB; and was a consultant for Abbvie, Amgen, BMS, Janssen, Galderma, GSK, Leo, Lilly, Merck, Novartis, Paladin Labs Inc., Pfizer, Bausch-Health, Actelion, Celgene, Cipher, and UCB. Drs Mufti, Maliyar, and Georgakopoulos and Authors Bagit, Rankin, Le, and Rimke have no conflicts of interest to declare.
